# MiR-219a-5p Enriched Extracellular Vesicles Induce OPC Differentiation and EAE Improvement More Efficiently Than Liposomes and Polymeric Nanoparticles

**DOI:** 10.3390/pharmaceutics12020186

**Published:** 2020-02-21

**Authors:** Iñaki Osorio-Querejeta, Susana Carregal-Romero, Ana Ayerdi-Izquierdo, Imre Mäger, Leslie A. Nash, Matthew Wood, Ander Egimendia, M. Betanzos, Ainhoa Alberro, Leire Iparraguirre, Laura Moles, Irantzu Llarena, Marco Möller, Felipe Goñi-de-Cerio, Goran Bijelic, Pedro Ramos-Cabrer, Maider Muñoz-Culla, David Otaegui

**Affiliations:** 1 Multiple Sclerosis Unit, Biodonostia Health Institute, 20014 San Sebastián, Spain; inaki.osorio@biodonostia.org (I.O.-Q.); aegimendia@cicbiomagune.es (A.E.); ainhoa.alberro@biodonostia.org (A.A.); leire.iparraguirre@biodonostia.org (L.I.); laura.moles@biodonostia.org (L.M.); maider.munoz@biodonostia.org (M.M.-C.); 2 Spanish Network of Multiple Sclerosis, 08028 Barcelona, Spain; 3 Center for Cooperative Research in Biomaterials (CIC biomaGUNE), Basque Research and Technology Alliance (BRTA), Paseo Miramón 182, 20014 Donostia , San Sebastián, Spain; scarregal.ciberes@cicbiomagune.es (S.C.-R.); illarena@cicbiomagune.es (I.L.); mmoller@cicbiomagune.es (M.M.); pramos@cicbiomagune.es (P.R.-C.); 4 CIBER de Enfermedades Respiratorias (CIBERES), 28029 Madrid, Spain; 5 TECNALIA, Basque Research and Technology Alliance (BRTA), Mikeletegi Pasealekua 2, 20009 Donostia-San Sebastián, Spain; ana.ayerdi@tecnalia.com (A.A.-I.); goran.bijelic@tecnalia.com (G.B.); 6 Department of Physiology, Anatomy and Genetics, University of Oxford, Oxford OX1 3PT, UK; imre.mager@dpag.ox.ac.uk (I.M.); lesliealanna@hotmail.com (L.A.N.); matthew.wood@dpag.ox.ac.uk (M.W.); 7 Department of Biochemistry, Microbiology and Immunology (BMI), University of Ottawa, 75 Laurier Ave E, Ottawa, ON K1N 6N5, Canada; 8 Biotechnology Area, Gaiker Technology Centre, 48170 Zamudio, Spain; goni@gaiker.es; 9 Ikerbasque, Basque Foundation for Science, 48013 Bilbao, Spain; betanzos@gaiker.es

**Keywords:** multiple sclerosis, remyelination, myelin, neurodegeneration, central nervous system, drug delivery

## Abstract

Remyelination is a key aspect in multiple sclerosis pathology and a special effort is being made to promote it. However, there is still no available treatment to regenerate myelin and several strategies are being scrutinized. Myelination is naturally performed by oligodendrocytes and microRNAs have been postulated as a promising tool to induce oligodendrocyte precursor cell differentiation and therefore remyelination. Herein, DSPC liposomes and PLGA nanoparticles were studied for miR-219a-5p encapsulation, release and remyelination promotion. In parallel, they were compared with biologically engineered extracellular vesicles overexpressing miR-219a-5p. Interestingly, extracellular vesicles showed the highest oligodendrocyte precursor cell differentiation levels and were more effective than liposomes and polymeric nanoparticles crossing the blood–brain barrier. Finally, extracellular vesicles were able to improve EAE animal model clinical evolution. Our results indicate that the use of extracellular vesicles as miR-219a-5p delivery system can be a feasible and promising strategy to induce remyelination in multiple sclerosis patients.

## Highlights

- miR-219a-5p enriched EVs are more efficient than liposomes and polymeric nanoparticles inducing oligodendrocyte precursor cell differentiation.
- EVs are able to cross an in vitro model of the blood–brain barrier in an effective way.
- miR-219a-5p enriched EVs are able to improve EAE clinical evolution.

## 1. Introduction

Myelin is a lipid membrane formed by oligodendrocytes (OL) in the central nervous system (CNS). Myelin wraps axons giving them trophic support and allowing a correct transmission of nerve impulses. However, in several diseases myelin is damaged causing an incorrect nerve impulse transmission and an imbalance in the homeostasis of axons, which can lead to neurodegeneration. The most common demyelinating pathology is multiple sclerosis (MS), a chronic CNS disease in which myelin is damaged by an autoimmune attack. In brief, activated T cells migrate across the blood–brain barrier (BBB), and induce an autoimmune response against myelin [1]. Interestingly, in the first stages of the disease, myelin can be endogenously restored in a process called remyelination. To perform this task, oligodendrocyte precursor cells (OPC) proliferate, migrate to the lesions, differentiate to oligodendrocytes and finally extend myelin’s sheaths around axons [2]. Nevertheless, when the disease progresses, the remyelination capacity of OL decreases and tends to fail. The reasons why this occurs are still not clear but it is thought to be related with the absence, insufficient migration or poor differentiation of OPCs [3,4].

Nowadays there is a wide range of treatments focused on the attenuation of the immune response, but there is still no treatment that promotes the regeneration of myelin, and therefore alternative strategies are appearing. The detection of undifferentiated OL in demyelinating lesions suggests that the stimulation of their differentiation process might be a feasible and potential way of inducing remyelination in MS patients [5].

With regard to this, several microRNAs have appeared as OPC differentiation mediators. MicroRNAs are small non-coding RNAs formed by about 22 base pairs with regulatory functions. More concretely, miR-219a-5p has been widely used in experimental analysis demonstrating that it is able to generate OPC differentiation and subsequent remyelination [6,7,8,9,10,11]. However, the administration of microRNAs to the CNS is difficult and so far, only invasive administration methods have been tested in animal models [9,12]. Although these experiments showed positive results, direct administration of microRNA to the CNS is not feasible for the treatment of MS patients. In order to administer the treatment in a non-invasive and efficient way, several delivery systems have been developed. The idea behind this strategy is to effectively dispense the microRNA to the CNS non-invasively and in a controlled manner, inducing OPC differentiation and remyelination.

In this work we have quantified and compared the ability as microRNA carriers of two synthetic and clinically approved drug delivery vectors (poly lactic-co-glycolic acid (PLGA) nanoparticles and liposomes [13]) versus biologically engineered extracellular vesicles (EVs) (which hold great promise as drug carriers for medical applications [14,15]), assessing their ability to induce OPC differentiation in a primary oligodendrocyte precursor cell culture. The capacity of these delivery systems to cross the blood–brain barrier (BBB) has also been analyzed. Finally, the Experimental Autoimmune Encephalomyelitis animal model has been used to study the remyelination potential of EVs, revealed as the most promising delivery system of the tested ones.

## 2. Materials and Methods

### 2.1. MicroRNA Carriers Synthesis and Loading 

**Figure 1:**
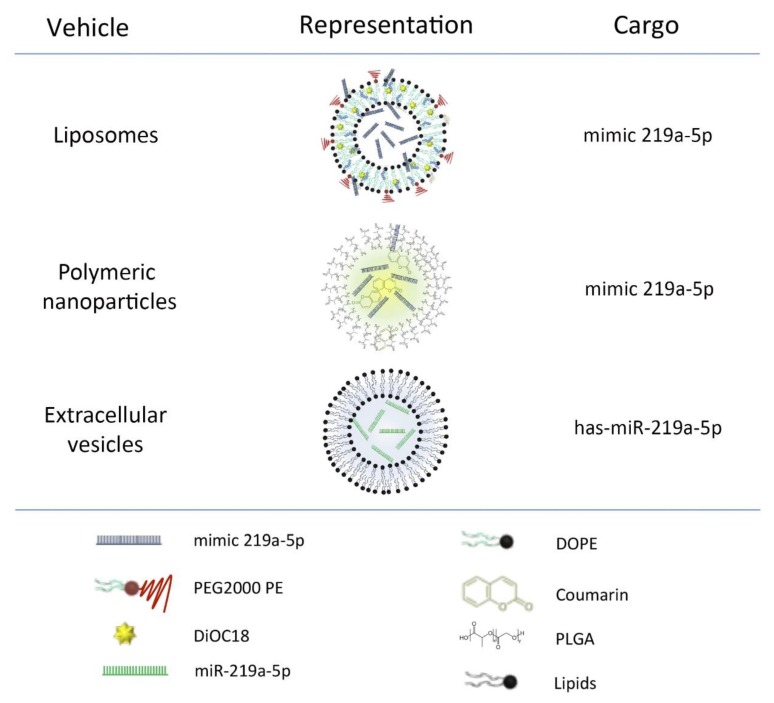
Schematic representation of liposomes, polymeric nanoparticles and extracellular vesicles. Carriers with their main components and the type of miR219a-5p they were loaded with. Liposomes and polymeric nanoparticles were loaded with synthetic microRNA. In contrast, extracellular vesicles (EVs) contained biologically produced microRNA. Polymeric nanoparticles and liposomes were also loaded with a dye (coumarin and DiOC18, respectively) to perform up-take studies.

A schematic representation of the main components of the carriers can be found in Figure 1.

#### 2.1.1. Liposomes

MiRIDIAN microRNA mimic mmu-miR-219a-5p (mimic-219a-5p) and miRIDIAN microRNA mimic Transfection Control with Dy547 (mimic-Red) (#C300576 and #CP004500 Dharmacon Inc, Lafayette, CO, USA) enriched liposomes or empty liposomes were prepared by the lipid film rehydration method [16,17]. Mixtures of lipids (typically 10 µmol) were prepared in a 6:1 *v/v* mixture of chloroform: methanol. The final mixtures contained 3ß-[*N*-(*N*’,*N*’-dimethylaminoethane)-carbamoyl]cholesterol (DC-Chol, molar fraction: x = 0.167), 1,2-dioctadecanoyl-sn-glycero-3-phosphocholine (DSPC: x = 0.617) and 1,2-distearoyl-sn-glycero-3-phosphoethanolamine-*N*-[amino(polyethyleneglycol)-2000] (ammonium salt) (PEG-DSPE: x = 0.05) (all from Avanti Polar Lipids, Alabaster, AL, USA). Some liposome formulations were fluorescently labelled by adding 13 µL of 3,3′-Dioctadecyloxacarbocyanine perchlorate (DiOC-18) dissolved in chloroform (1mg/mL) to the lipid mixture before the lipid film formation. Lipid films were formed by evaporation of chloroform: methanol on a rotavapor operated under vacuum at 30 °C, followed by 2 h drying under nitrogen flow. Lipid films were hydrated with 2 mL of RNAse-free water at 65 °C. MiRNAs were added in a ratio of miRNA:lipid of 0.9 nmol miRNA:µmol lipid. The lipid film with the miRNA was allowed to hydrate overnight at 4 °C forming miRNA-PEgylated liposome complexes. After that, the liposome dispersions were diluted twice with water and extruded 16 times at 65 °C through polycarbonate membrane filters (Whatman, PLC, Rentfort, UK) of decreasing pore diameter (400, 200, and 80 nm). After this process, miR-219a-5p-liposomes (219-Lp), mimic-Dye-547-liposomes (Red-Lp) and empty liposomes (E-Lp) were generated (Figure 1).

#### 2.1.2. Polymeric Nanoparticles

Poly lactic-*co*-glycolic acid (PLGA) nanoparticles were synthesized using water in oil in water (*w/o/w*) double emulsion technique. In brief, miRIDIAN microRNA mimic mmu-miR-219a-5p or miRIDIAN microRNA mimic Transfection Control with Dy547 were dissolved in 200 µL of surfactant (0.5% poly vinyl alcohol (PVA) as an excipient), and were homogenized with 10% *w/v* PLGA in dichloromethane with a Misonix sonicator probe (Misonix, Inc., Farmingdale, NY, USA) at 10 W for 3 min. This initial water in oil emulsion (*w/o*) was then added to 15 mL of 5% *w/v* solution of PVA for a second emulsion step and was homogenized at 12 W for 3 min. The resultant water in oil in water (*w/o/w*) double emulsion was then subjected to organic solvent evaporation under stirring at 600 rpm overnight at room temperature. The sample was centrifuged in water, 4 times at 14,000 rpm, to wash out any water-soluble surfactant and polymer residues. Finally, miR-219a-5p-nanoparticles (219-Np), mimic-Dye547-nanoparticles (Red-Np), and empty nanoparticles (E-Np) were generated (Figure 1).

#### 2.1.3. Extracellular Vesicles

pLKO.1 lentiviral particles containing miR-219a-5p sequence or empty particles were infected in HEK293T cells. Selection was made by culturing the cells with puromycin (Cayman Chemical, Pittsfield, MA, USA) and cells were maintained with this antibiotic for the rest of the experiment to avoid the growth of non-infected cells. To isolate EVs, cells were grown to an 80% confluence in media (DMEM Thermo Fisher, 10% FBS Thermo Fisher and 1% Puromycin) followed by a change in the media to OptiMEM (Thermo Fisher, Waltham, MA, USA) containing puromicyn for 36 h. Then, media was recollected and centrifuged at 2.000 g for 10 min to pellet cell debris. Supernatant was used to concentrate EVs using Tangential Flow Filtration system with VivaFlow 50R 10.000 MW membrane (Sartorious, Goettingen, Germany) followed by the use of centrifugal filters of 10.000 MW (Merck Millipore, Burlington, MA, USA). Afterwards, EVs were isolated by differential centrifugation steps. Briefly, the sample was centrifuged at 13.000 g for two minutes, supernatant recollected and centrifuged again at 20.000 g for 20 min. Then, pellet was recollected and resuspended in dPBS. With this process miR-219a-5p enriched extracellular vesicles (219-EVs) and non-enriched extracellular vesicles (Ne-EVs) were obtained. A Bradford assay was performed to quantify the protein levels of the samples, which was used to standardize EVs administration.

In order to obtain fluorescent EVs for up-take and BBB crossing experiments, Ne-EVs were labelled with CM-Dil (Celltracker CM-DiI, #C7001, Thermo Fisher) as previously described [18], obtaining Ne-EVs-CM-Dil. In short, EVs were incubated with 1 µg/mL of Celltracker CM DiI for 5 min at 37 °C and 15 min at 4 °C. Then the sample was centrifuged at 20.000 g for 20 min in order to pellet EVs and remove the excess of dye. EVs were resuspended in dPBS (Figure 1).

### 2.2. Characterization of microRNA Carriers

#### 2.2.1. Nano Tracking Particle Analysis (NTA)

NTA was performed in a NanoSight LM10 device (Malvern Panalytical, Malvern, UK) as previously described [19]. In brief, samples were diluted in filtered dPBS to get accurate acquisition (200–900 recorded tracks). Camera settings were fixed and maintained for all samples (gain: 6.9; camera level: 5). For each sample, two videos of 1 min were recorded and analyzed with NanoSight NTA software 3.2 (Malvern Panalytical). Total particles counts were obtained and profile distribution graphs generated.

#### 2.2.2. Droplet Digital PCR

RNA encapsulated in liposomes, polymeric nanoparticles and EVs was isolated using Trizol LS protocol following the manufacturer’s instructions (#10296028, ThermoFisher). For EVs the RNA concentration was quantified by NanoDrop ND-1000 spectrophotometer. Levels of RNA in liposomes and polymeric nanoparticles were not quantified by NanoDrop ND-1000 due to the low yields of total RNA in the sample (only miRIDIAN microRNA mimic-219a-5p was present). RNA was reverse transcribed using TaqMan™ MicroRNA Reverse Transcription Kit (# 4366596; Thermo Fisher) following the manufacturer’s protocol in a Veriti Thermal Cycler (Applied Biosystems, Foster City, CA, USA). miR-219a-5p expression analysis was performed using miRNA TaqMan Assay (ID: 522, Thermo Fisher) and ddPCR Supermix for probes (no dUTP) (#186-3023, BioRad Laboratories, Hercules, CA, USA) in a QX200 droplet digital PCR system (BioRad). Analysis was carried out using QuantaSoft 1.6.6 software (BioRad). In order to compare results between vehicles, the number of copies of miR-219a-5p per particles (quantified by NTA) were described.

#### 2.2.3. CryoTEM

Liposomal, polymeric nanoparticle and extracellular vesicle solutions were vitrified following standard protocols described elsewhere [20]. Quantifoil holey carbon film grids (Orthogonal Array of 2 µm Diameter Holes—2 µm Separation, mounted on a 300 M Cu grid, #657-300-CU, Ted Pella) were vitrified in liquid ethane in Vitrobot (FEI) after a negative glow-discharged treatment of the grids and deposition of 3 µL of each sample. Cryo-transfer sample holders of type GATAN Model 626 kept the sample vitrified during electron microscopy analysis. Every sample was observed on a JEM-2100F UHR (80-200kV, JEOL Ltd. Akishima, Tokio, Japan) field emission gun (FEG) transmission electron microscope at different magnifications (8000× and 30,000×). Low-dose micrographs were recorded on a state of the art TVIPS F216 CMOS camera (2k × 2k).

#### 2.2.4. MicroRNA Microarray

Following manufacturer’s instructions, total RNA (200 ng) was labelled using the FlashTag Biotin labelling kit (Ref: 901911; Thermo Fisher) and hybridized to the GeneChip miRNA 4.0 Array (Ref: 902412; Thermo Fisher), which covers 1908 mouse miRNAs. Briefly, RNA molecules were polyadenylated and a biotin-labelled DNA molecule was attached in a subsequent ligation step. Finally, labelled RNA was hybridized to the array, washed and stained in a GeneChip Fluidics Station 450 and scanned in a GeneChip Scanner 7G (Affymetrix, Santa Clara, CA, USA).

### 2.3. Oligodendrocyte Precursor Cell Culture

The isolation of OPCs was carried out as previously described [21]. Briefly, postnatal P1-P3 C57BL/6 mice were sacrificed by decapitation. Once the brain was removed, meninges were extracted and tissue digested mechanically and enzymatically with papain at 37 °C for 14 min. Twenty-five microliters of OPC medium (DMEM (#041966; Thermo Fisher), supplemented with 10% Fetal Bovine Serum (# 16000044; Thermo Fisher) and 1% Antibiotic-Antimycotic (#A5955; Sigma, Santa Luis, MO, USA) was added to the sample. Then, 300 µL of a DNase solution (1%, #DN-25; Sigma) was added to stop papain digestion. After a centrifugation step at 190 g for 10 min, supernatant was removed and the pellet, after resuspension in 1 mL of OPC media, was filtered through a 100-µm nylon mesh strainer (Falcon, Seaton Delaval, UK). Finally, up to 8 mL of OPC media were added to the cells which were seeded in a 75-cm^2^ Poly-l-Ornithine coated flask. After one week of incubation at 5% CO_2_ and 37 °C, renewing the media every 2–3 days, the culture was shaken for 15 h at 200 rpm and one extra hour at 250 rpm. This was followed by media collection, which was passed through a 40-µm nylon cell strainer (Falcon) and centrifuged at 190 g for 10 min. The pellet was resuspended in 10 mL of OPC media. The suspension was plated on an untreated plastic Petri dish at 37 °C for one hour to allow the microglial cells to attach to the Petri dish. Then, the media was collected, centrifuged at 190 g for 10 min, filtered again through a 40-µm strainer and incubated in a new uncoated Petri dish for 30 min. After the 30 min, the media was collected, and centrifuged at 190 g for 10 min to finally obtain the purified OPCs. A total of 20.000 cells were incubated on a laminin-treated plate to perform uptake and differentiation experiments.

#### 2.3.1. Up-take Studies

Note: The fact that each vehicle has to be generated under different protocols made it impossible for us to administer them in the same volume and concentration. To solve that, we added each vehicle in order to dispense similar particles counts, independently of the volume that was added to the culture. Red-Lp, Red-Np labelled with DiOC18 and Coumarin respectively and Ne-EVs-CM-Dil were added to the OPCs and incubated for 24 h in order to determinate the percentage of cells that were up-taking the particles. A total volume of 100 µL of liposomes, 1 µL of polymeric nanoparticles, and 10 µL of EVs (100 μg of quantified protein) were administered to the culture. These volumes made a final number of 2.7 × 10 ^5 to 6^ particles for all vehicles.

Twenty-four hours later, OPCs were fixed with 4% paraformaldehyde and labelled with Hoechst. Images were obtained with 20x magnification in a Nikon Eclipse80i (Nikon, Minato, Japan) and NIS elements AR 3.2 (Nikon) was used to capture the images. Fields were randomly selected. LSM 880 confocal microscopy (Zeiss, Oberkochen, Germany) was used at a 63× (oil, 1.4 NA), magnification objective to obtain individual cell images using 405, 488, 561 excitation lines. Z-stacks where acquired to characterized uptake, frame size was 1024 × 1024 pixels. Processing of images was performed with open source software ImageJ. Positive and negative cells were counted and the average up-take levels quantified.

#### 2.3.2. Differentiation Studies

Similar to up-take studies, 219-Lp, E-Lp, 219-Np, E-Np, 219-EVs, and Ne-EVs were administered to OPCs in the following concentrations: A total volume of 100 µL of liposomes, 1 µL of nanoparticles and 10 µL of EVs (100 μg of quantified protein) were dispensed to the culture making a final concentration of 2.7 × 10 ^5 to 6^ per well. Due to the low quantity of miR-219a-5p present in the cargo of EVs, these vesicles were administered daily. A unique dose of liposomes and nanoparticles was administered at time zero. Three days after administration, RNA was isolated from OPC in order to perform gene expression studies and determine the degree of cell differentiation. RNA was isolated using Trizol LS protocol following the manufacturer´s instructions (#10296028 Thermo Fisher). Then, total RNA was reverse transcribed using High-Capacity cDNA Reverse Transcription Kit (#4368814, Thermo Fisher) following manufacturer’s protocol in a Veriti Thermal Cycler (Applied Biosystems). Gene expression analysis of chondroitin sulfate proteglycan (*Ng2*), platelet derived growth factor receptor alpha (*Pdgfra*), 2’,3’-cyclic nucleotide 3’ phosphodiesterase (*Cnpase*) myelin oligodendrocyte glycoprotein (*Mog*), myelin basic protein (*Mbp*) and proteolipid protein (*Plp1*) were performed in order to establish the differentiation degree of the cells, using KiCqStart SYBR Green qPCR ReadyMix and KiCqStart SYBR Green Primers (KCQS02 and KSPQ12012, Sigma). Ribosomal protein L13α (*Rpl13a*) and phosphoglycerate kinase 1 (*Pgk1*) were used as endogenous controls. CFX384 Thermal Cycler (Bio-Rad) was used to run the qPCR and raw data was processed in Bio-Rad CFX Manager software. The calculation of relative expression was carried out with Excel software using the 2^−DDCT^ method [22].

### 2.4. Blood–Brain Barrier Crossing Studies

bEnd.3 endothelial cell line and CTX astrocyte cell line were obtained from the American Type Cell Culture collection (ATCC). Cell lines were maintained in complete culture media according to the suppliers’ instructions, and were routinely grown as a monolayer in tissue culture grade flask. When cells exceeded 70% confluence (but less than 90%), they were sub-cultured on new culture flasks. All cell cultures were maintained in standard cell culture conditions (37 °C, 5% CO_2_, and 95% humidity). In co-cultures experiments, the endothelial cell line was seeded on the top of 24 well semipermeable transwell inserts (6 × 10^4^ cells/cm^2^) previously pre-coated with rat tail collagen and co-cultured with CTX cell line.

Monolayer integrity assessment was done by measuring transendothelial electrical resistance (TEER). TEER measurements were taken manually every following day until a steady state was reached using an EVOM2 Epithelial Volt/Ohmmeter connected to a pair of chopstick electrodes STX2 (World Precision Instruments, Berlin, Germany). To calculate the TEER value of each insert, the average resistance values of three cell free coated inserts was subtracted from the resistances obtained in the presence of the endothelial cells. This value was then multiplied by the effective surface area of the filter (0.33 cm^2^). The TEER values (Ω*cm^2^) were used to determine the correct development of the BBB in vitro model.

#### Crossing Studies

Confluent pre-coated cell culture inserts containing bEnd.3 cells were washed with pre-warmed Ringer-HEPES buffer (RHB) and then transferred to new 24-well plates containing 0.8 mL/well pre-warmed RHB buffer. At the beginning of the experiment, 200 µL of Red-Lp at 5.7 µg/mL, 57 µg/mL, 0.42 mg/mL mM and 0.84 mg/mL, Red-NP at 0.1 mg/mL and 0.5 mg/mL or Ne-EVs-CM-Dil at 0.1 mg/mL, and 0.5 mg/mL were added to the donor chamber (apical side) and inserts were incubated for 15, 30, 45, and 60 min in an orbital shaker at 37 °C. The permeability of the vehicles was measured in the apical-to-basolateral direction. At each incubation time, the inserts were transferred to new 24-well plates previously replenished with 0.8 mL/well of fresh, pre-warmed RHB buffer. Permeability was also measured on cell-free coated inserts. Fluorescence intensity was quantified by fluorescence measurement.

Permeability coefficient (Pe) of the endothelial monolayer was calculated using the following equation to obtain a concentration-independent permeability value:

$$\frac{1}{PS}=\frac{1}{me}-\frac{1}{mf}\;Pe=\frac{PS}{s}$$

where *PS* is the permeability x surface area product (0.33 cm^2^) and *me* and *mf* are the slopes of the clearance curves corresponding to the endothelial cells grown on filters and to the filters without cells, respectively. The permeability coefficient is the result of dividing the *PS* by the surface of the transwell insert. Each condition was tested in triplicate in each experiment. Values are given as mean ± standard deviation (SD).

Finally, after permeability studies, lucyfer yellow (LY) analysis was also performed as mentioned above to determine if liposomes, nanoparticles and EVs interactions with microvascular endothelial cells alter the barrier permeability (toxicity).

### 2.5. Experimental Autoimmune Encephalomyelitis (EAE)

All animal procedures were approved by the Biodonostia Health and Research Ethics Committee (CEEA17_002; 13-02-2017), and local authorities.

#### 2.5.1. Induction and Clinical Evaluation

Ten-week-old C57BL/6 female mice were immunized with Hooke Kit^TM^ MOG_35–55_/CFA Emulsion PTX kit (#EK2110, Hooke Laboratories, Lawrence, MA, USA) as described in the manufacturer’s protocol. Briefly, animals were anesthetized with isoflurane 2% and 100 µL of MOG_35–55_ and Freund’s adjuvant emulsion were injected subcutaneously in two locations, administrating a total volume of 200 µL per animal. Two- and twenty-four hours after emulsion administration, 80 ng of Pertussis Toxin were administered to each animal. Animals were weighed and scored daily following Hooks labs clinical evaluation scale. The monitoring was performed in a blind manner in order to achieve objective scores.

#### 2.5.2. Extracellular Vesicles Administration

One hundred micrograms of 219-EVs and Ne-EVs were intranasally administered 2 and 8 days after disease induction. To do this, mice were anesthetized with isoflurane 2% and 10 µL of EVs (10 μg/μL) were administered to each animal in four doses of 2.5 µL, two per nostril, for 10 min.

#### 2.5.3. Sample Extraction

Animals were anesthetized with isoflurane 2% and cardiac puncture was carried out to collect blood at final term. Additionally, spinal cords were extracted and fixed in PFA at 4% for 72 h. Then, spinal cords were stored at 4 °C in PBS with 0.01% of azide until ex-vivo high resolution Magnetic Resonance Imaging was carried out.

#### 2.5.4. Plasma Derived Cytokine Measurement

Blood samples obtained from cardiac puncture were centrifuged at 300 *g* for 15 min and plasmas were isolated. Plasma concentration of IL-10 IL-17A and TNFα were measured by a mouse high sensitivity T cell magnetic bead panel as previously described and following the manufacture’s protocol (MHSTMAG-70K Millipore) [23]. Then Bio-Plex MagPix (MerckMillipore, Burlington, MA, USA) was used to run the samples and perform the analysis.

#### 2.5.5. Magnetic Resonance Imaging

Magnetic Resonance Images (MRI) of spinal cords of mice (animals with lowest clinical score per group; *n* = 1) were acquired using a Bruker Biospec USR 117/16 MRI system (Bruker Biospin GmbH, Ettlingen, Germany) interfaced to 4 transmit and 8 receive RF channels with a XYZ set of actively shielded gradients 750 mT/m, and a slew rate of 6660 T/m/s. RF transmission and reception was achieved by using a transmit volumetric coil of 72 mm of i.d. (T1148V3), and receive surface coil (mouse brain surface coil, T11657V3) of approx. 2 cm diameter, both from Bruker Biospin GmbH (Ettlingen, Germany). 

Tissue samples were immersed in PBS for imaging. After the acquisition of a series of scout images, Diffusion Weighted Images of the samples were acquired using a DtiStandard sequence for Bruker software Paravision 6.0.1, with the following parameters: Spin-Echo DWI, 12 diffusion directions (d = 4 ms, D = 11 ms, b = 1000 s mm^−2^), 1 image with b = 0 s mm^−2^, echo time TE = 21 ms, repetition time TR = 3000 ms, N Averages = 2, Image Matrix of (256 × 256) points with a Field-of view FOV = (25.6 × 25.6 mm), giving an in-plane resolution of (100 × 100 μm), and acquiring 60 consecutive slices of 0.2 mm thickness. Spectrometer bandwidth was set to BW = 66 kHz and the total scanning time resulted in 4 h 9 m 36 s. The open source software DIPY (Diffusion Imaging in python), with local PCA denoising, was used for the processing of acquired DTI images, to obtain fractional anisotropy (FA) Further image analysis was performed with Image-J software, from NIH.

### 2.6. Statistical Analysis

qPCR data relative expression was calculated by 2 ^−DDCT^ method. Unpaired t-test and one factor analysis of variance (ANOVA) with Bonferroni-Dunn’s correction were performed to assess differences in BBB crossing analysis. Mann Whitney test was used to analyze differences in the score of EAE animals and the significance of MRI fractional anisotropy of MRI images.

## 3. Results and Discussion

### 3.1. Characterization of microRNA Carriers

**Figure 2:**
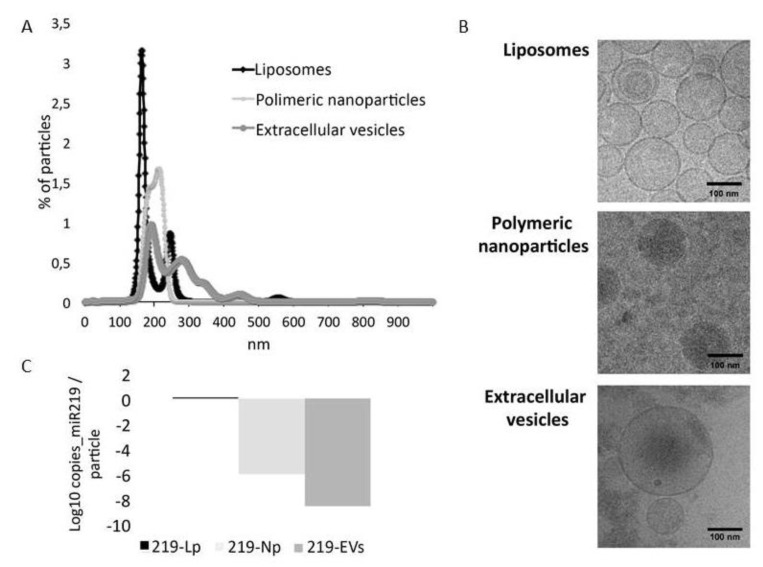
Vehicles characterization: (**A**) Nanoparticle Tracking Analysis (NTA) of liposomes, polymeric nanoparticles and extracellular vesicles. (**B**) Transmission electron cryomicroscopy images of the three vehicles showing correlation in size with NTA. (**C**) Levels of miR-219a-5p in liposomes (219-Lp), nanoparticles (219-Np) and Extracellular vesicles (219-EVs). Liposomes are the most enriched vehicles followed by nanoparticles and finally EVs.

NTA was used to characterize the distribution in size and number of particles present in the samples. As shown in Figure 2A, liposomes showed two main populations, the principal one at a size of 160 nm and a secondary one at 230 nm. Polymeric nanoparticles displayed a more homogeneous size distribution the main size being between 180 and 220 nm. In contrast, EVs were the most heterogeneous sample with particles ranging from 150 to 400 nm. These results are in line with the genesis procedure of each vehicle. On the one hand, liposomes and polymeric nanoparticles are synthetically generated in the laboratory with standard protocols to produce a homogeneous sample. On the other hand, extracellular vesicles are membrane-bound particles secreted by cells. They have been described to be different in size depending on their biogenesis [24]. However, it must be noted that in many scenarios it can be difficult to separate those based exclusively on their size [25] indicating that our sample is a heterogeneous sample of EVs (exosomes and microparticles). These results were confirmed by cryogenic electron microscopy in which similar size vesicles can be found when compared to NTA (Figure 2B).

Regarding the amount of microRNA carried in each vehicle, we determined that liposomes encapsulate miR-219a-5p more efficiently than polymeric nanoparticles, and these more than EVs. With particle counts data obtained from NTA, we quantified the copies of microRNA per particle, determining that liposomes were the most enriched particles followed by PLGA nanoparticles and finally extracellular vesicles (Figure 2C). In our hands, EVs were the most time-consuming vehicle to be generated since HEK293T cells need approximately 2 weeks to be grown, and two additional days are required to isolate the EVs from them. On the other hand, synthesis of liposomes and polymeric nanoparticles are less time consuming and more reproducible.

### 3.2. Liposomes and Polymeric Nanoparticles are More Efficiently Taken Up by OPCs. Extracellular Vesicles Induce Differentiation More Efficiently

**Figure 3:**
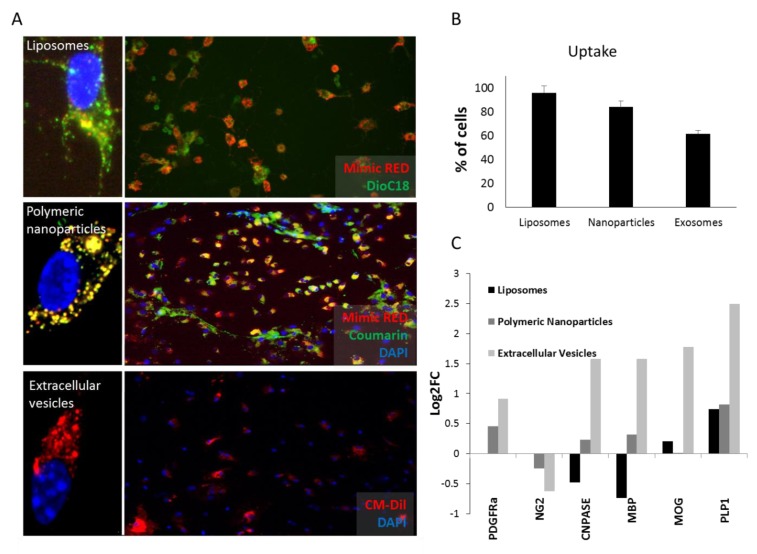
Up-take and differentiation levels of oligodendrocyte precursor cells (OPCs) after vehicles administration. (**A**) Uptake studies of liposomes, polymeric nanoparticles and extracellular vesicles. Liposomes containing 3,3′-Dioctadecyloxacarbocyanine perchlorate dissolved in chloroform (1mg/mL) (DOiC)18 (green) and mimic-Red (red), nanoparticles containing Coumarin (green) and mimic-Red (red) and EVs labelled with CM-DiL (red) are shown. Dapi (blue) was used to stain nucleus. Individual cells are imaged by confocal fluorescence microscopy of representative samples. (**B**) Percentage of OPCs that are able to take up each vehicle. Liposomes are the most efficient vehicle followed by polymeric nanoparticles and finally EVs. (**C**) Expression levels (expressed as de logarithm 2 of the fold change; Log2FC) of myelin related genes in OPC cultures treated with each vehicle compared to the same empty vehicle. EVs are the only vehicle able to induce OPC differentiation. Note the lack of PDGFRa and NG2 data in liposomes due to technical problems.

Next, we tested the capacity of OPCs to uptake the three nanosystems by labelling liposomes, polymeric nanoparticles and EVs with DiOC18, Coumarin or CM-DiI, respectively. Additionally, liposomes and polymeric nanoparticles were loaded with miRIDIAN microRNA mimic Transfection Control with Dy547 (mimic-Red) in order to see the presence of the microRNA in the cells. 24 h after administration, cells were fixed and microscopy images acquired revealing that liposomes were more efficiently uptaken by cells (95.83%, Stdv 5,9), followed by nanoparticles (83.98%, Stdv 5,1) and finally EVs (61.48%, Stdv 2,8) (Figure 3A,B). To study the potential of the three vehicles to induce OPC differentiation, a necessary step for remyelination, 219-Lp, 219-Np, and 219-EVs were compared to their respective empty or non-enriched vehicles. Interestingly we observed that only 219-EVs were able to significantly increase the expression of myelin related genes (*Cnpase*, *Mbp*, *Mog*, and *Plp1*) without modifying OPC related genes (*Pdgfra* and *Ng2*) indicating a more differentiated state of the OPCs (Figure 3C). Neither liposomes nor nanoparticles, were able to induce significant OPC differentiation.

Liposomes and polymeric nanoparticles were expected to be the most appropriate microRNA delivery systems due to their homogeneity in composition and cargo together with a controlled generation process. Nevertheless, although EVs showed the lowest miR-219-5p and up-take levels when compared to liposomes and nanoparticles it was the only vehicle able to induce OPC differentiation. EVs are biologically formed vesicles, which play an essential role in indirect intercellular communication and that can transfer not only a wide range of miRNAs but RNA binding proteins (such as Dicer, Ago2 or TRBP [26,27]) cytosolic proteins, lipids, metabolites and genetic material which can be delivered to recipient cells, [28,29]. In relation to this, EVs have shown to be efficient microRNA delivery systems for several diseases [30]. This opens an interesting discussion about the potential role that other factors may play as facilitators of the effect produced by miR-219a-5p. In fact, our results highlight the importance of the biological complexity of EVs for OPC differentiation, complexity that is achieved in neither liposomes nor polymeric nanoparticles. We next investigated which other miRNAs might support the function of EVs to promote OPC differentiation by using microarray technology. Surprisingly, miR-219a-5p was not detected by this array. This unexpected result questions the sensitivity of microarray technology, given that we had already confirmed the overexpression of miR-219a-5p in these EVs. Nonetheless, to be sure that miR-219a-5p was present in enriched EVs, we performed a qPCR experiment, which confirmed our previous ddPCR data (Ct value in qPCR = 27). Additionally, both ddPCR and qPCR demonstrated that 219-EVs contained between 30 and 100 times more miR-219a-5p than Ne-EVs.

### 3.3. Extracellular Vesicles Cross the BBB More Efficiently than Liposomes and Nanoparticles

In an attempt to go a step further, we decided to study the capacity of each vehicle to cross the BBB. Firstly, we ensured the effective establishment of a working BBB in the in vitro model, by performing TEER measurements, which reflect the blockage to the movement of small ions through the cell monolayers. TEER values were monitored from the day after seeding the endothelial cells, observing an increase with culture time, and reaching their maximum values after 3–7 days. The glial effect on the endothelial TEER increase was noticeable 3 days after bEND.3 seeding on Transwell filters.

**Figure 4:**
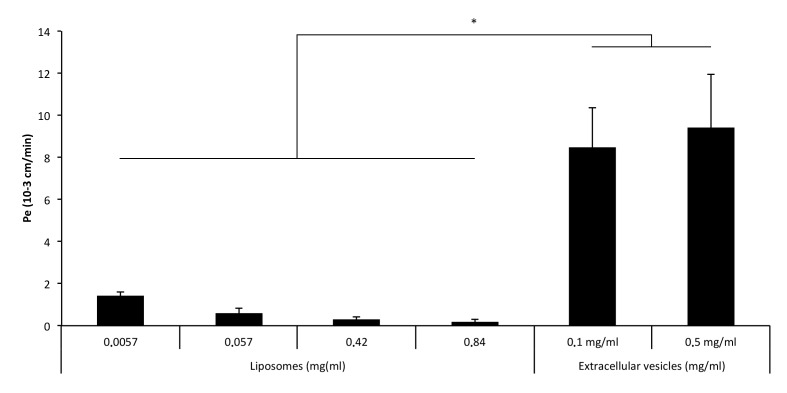
Transendothelial migration of liposomes and exosomes: Permeability of endothelial monolayer for liposomes and extracellular vesicles. Polymeric nanoparticles were not able to cross the blood–brain barrier (BBB) and are not, therefore, shown in the figure. EVs showed the highest levels of permeability, indicating that could be a proper candidate for microRNA delivery to the central nervous system (CNS). (*) *p* < 0.005 with respect to Liposomes values.

As a reflection of tight junction functionality, the permeability (Pe) of endothelial monolayers was tested for Red-Lp, Red-Np, and Ne-EVs-CM-Dil. Regarding the different experimental conditions, a statistically significant reduction in the paracellular permeability for liposomes was observed at increasing concentrations (Figure 4). Nanoparticles showed values close to zero, indicating that they were not able to cross the BBB according to the used in vitro model. Interestingly, EVs showed the highest Pe values, indicating that they were very efficient in crossing the BBB.

Subsequently, LY permeability was analyzed determining no alteration in the monolayer permeability by Red-Lp, Red-Np, and Ne-EVs-CM-Dil indicating the absence of toxicity and that they did not alter the monolayer permeability.

### 3.4. MiR-219a-5p Enriched Extracellular Vesicles Improve EAE Clinical Evolution

**Figure 5:**
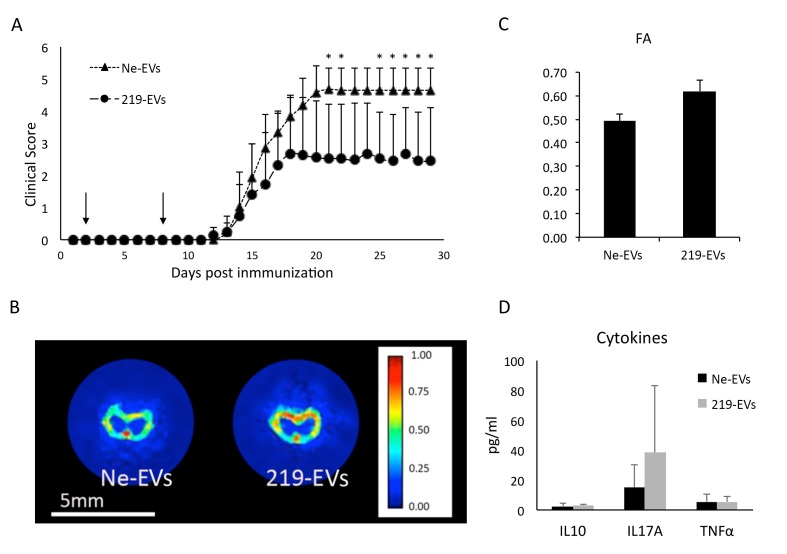
Intranasal administration of miR-219a-5p enriched exosomes promotes myelin regeneration. (**A**) Clinical evaluation of animals treated with non-enriched extracellular vesicles (Ne-EVs) and with miR-219a-5p enriched extracellular vesicles (219-EVs) (100 μg of EVs per dose; animals received two doses at days 2 and 8 after disease induction). 219-EVs treated animals showed a significant decrease in the clinical evaluation after the disease peak (*n* = 4). (**B**) MRI of spinal cord of a Ne-EVs treated animal and a 219-EVs treated mouse showing the fractional anisotropy (FA). (**C**) FA values of a section of the spinal cord of previous animals showing a decrease in FA values when treatment was Ne-EVs, indicating that remyelination is occurring. (**D**) No significant differences between both groups in pro-inflammatory cytokines were found, indicating that the effect induced by EVs was not related to an anti-inflammatory process.

Once established that EVs were the only vehicle able to induce a significant OPC differentiation, we focused on them to test their ability to improve the clinical score in the EAE murine model. EVs were intranasally administered as previously reported [31], to allow them to reach the CNS. Two doses were dispensed at days 2 and 8 after disease induction and the clinical score was monitored daily for 29 days, as detailed in the methods section. We detected significant differences between 219-EVs versus Ne-EVs treated group, at days 21, 22, and 25–29. Interestingly, differences appeared after the disease peak, indicating that EVs might be promoting myelin regeneration, instead of reducing or slowing down degeneration (Figure 5A). To confirm this, *ex-vivo* MRI images were obtained from spinal cords of mice from both groups (Figure 5B). Fractional anisotropy, a measure of the linearity of the diffusion (being its value between zero and one (zero = isotropic diffusion; one = diffusion occurs only along one axis)) is significantly increased in miR-219a-5p enriched EVs treated animals when compared to non-enriched EVs treated animals (*p* = 0.001; Figure 5C).

Additionally, we analyzed the inflammatory patterns in plasma of EAE mice, but no significant differences were found in the analyzed plasma derived cytokine levels between both groups (Figure 5D). This result suggests that 219-EVs do not regulate the immune system of the animal as expected, supporting the idea that significant differences shown in the clinical score evolution may be related to myelin regeneration.

## 4. Conclusions

From our studies, several insights in the therapeutical use of miR-219a-5p were obtained. First, liposomes and PLGA nanoparticles were able to entrap higher amounts of miR-219a-5p and showed higher uptake levels than EVs. In contrast, EVs were surprisingly the only delivery system able to induce a significant OPC differentiation. We must remember that EVs are biological delivery systems that contain other proteins, lipids, and genetic material, apart from RNA, which can be integrated into the cell in several ways, and that contain microRNA-processing molecules. This indicate that the efficiency of EVs as microRNA delivery systems could be influenced by their biological complexity, making them more efficient delivery systems than DPSC based liposomes or PLGA nanoparticles. Additionally, EVs showed the highest BBB permeability levels, making them the most promising microRNA delivery system.

Furthermore, intranasally administered miR-219a-5p enriched EVs successfully decreased clinical scores in the EAE model, without affecting the tested anti-inflammatory pathways. In addition, significant differences in clinical score were seen after the disease peak, indicating that EVs might be increasing the myelin production.

In summary, miR-219a-5p has been identified as a necessary but not sufficient condition to induce remyelination in the EAE model. Moreover, miR-219a-5p enriched EVs stimulate OPC differentiation, cross the BBB and improve EAE clinical evolution opening a therapeutic approach for MS patients. In addition, this work shows that the use of EVs as a microRNA delivery system for CNS diseases can be a promising and feasible tool.
